# *Wolbachia* Interferes with Ferritin Expression and Iron Metabolism in Insects

**DOI:** 10.1371/journal.ppat.1000630

**Published:** 2009-10-23

**Authors:** Natacha Kremer, Denis Voronin, Delphine Charif, Patrick Mavingui, Bertrand Mollereau, Fabrice Vavre

**Affiliations:** 1 Université de Lyon, Lyon; Université Lyon 1; CNRS, UMR 5558, Laboratoire de Biométrie et Biologie Evolutive, Villeurbanne, France; 2 Université de Lyon, Lyon; Université Lyon 1; CNRS, UMR 5557, Laboratoire d'Ecologie Microbienne, Villeurbanne, France; 3 LBMC, UMR5239 CNRS/Ecole Normale Supérieure de Lyon, IFR 128 Biosciences Lyon Gerland, Université de Lyon, Lyon, France; Stanford University, United States of America

## Abstract

*Wolbachia* is an intracellular bacterium generally described as being a facultative reproductive parasite. However, *Wolbachia* is necessary for oogenesis completion in the wasp *Asobara tabida*. This dependence has evolved recently as a result of interference with apoptosis during oogenesis. Through comparative transcriptomics between symbiotic and aposymbiotic individuals, we observed a differential expression of ferritin, which forms a complex involved in iron storage. Iron is an essential element that is in limited supply in the cell. However, it is also a highly toxic precursor of Reactive Oxygen Species (ROS). Ferritin has also been shown to play a key role in host–pathogen interactions. Measuring ferritin by quantitative RT-PCR, we confirmed that ferritin was upregulated in aposymbiotic compared to symbiotic individuals. Manipulating the iron content in the diet, we showed that iron overload markedly affected wasp development and induced apoptotic processes during oogenesis in *A. tabida*, suggesting that the regulation of iron homeostasis may also be related to the obligate dependence of the wasp. Finally, we demonstrated that iron metabolism is influenced by the presence of *Wolbachia* not only in the obligate mutualism with *A. tabida*, but also in facultative parasitism involving *Drosophila simulans* and in *Aedes aegypti* cells. In these latter cases, the expression of *Wolbachia* bacterioferritin was also increased in the presence of iron, showing that *Wolbachia* responds to the concentration of iron. Our results indicate that *Wolbachia* may generally interfere with iron metabolism. The high affinity of *Wolbachia* for iron might be due to physiological requirement of the bacterium, but it could also be what allows the symbiont to persist in the organism by reducing the labile iron concentration, thus protecting the cell from oxidative stress and apoptosis. These findings also reinforce the idea that pathogenic, parasitic and mutualistic intracellular bacteria all use the same molecular mechanisms to survive and replicate within host cells. By impacting the general physiology of the host, the presence of a symbiont may select for host compensatory mechanisms, which extends the possible consequences of persistent endosymbiont on the evolution of their hosts.

## Author Summary

*Wolbachia* are intracellular bacteria that infect numerous invertebrate species, where they are generally facultative for their host. Surprisingly, the wasp *Asobara tabida* is dependent on *Wolbachia* for egg production: in uninfected females, the cells necessary for egg maturation die prematurely as a result of apoptosis. When we analyzed the genetic basis of this dependence, we found that ferritin, a protein involved in the regulation of iron homeostasis, was over-expressed in uninfected individuals. We also found that *Wolbachia* interferes with iron metabolism and ferritin expression in other host–*Wolbachia* associations. Furthermore, *Wolbachia* itself responds to changes in iron concentration by changing the expression of bacterioferritin. Iron is in short supply within the cell, and is necessary for both host and symbiont; our findings highlight the key role of iron in host–symbiont interactions, as had previously been shown for host–pathogen interactions. Furthermore, iron homeostasis is involved in the regulation of oxidative stress, which in turn is involved in inducing cell death. *Wolbachia* could also interfere with iron in a way that limits oxidative stress and cell death, thus promoting its persistence within host cells. In *A. tabida*, we show that iron induced cell death in the ovaries, suggesting that iron metabolism could also be linked to the evolution of dependence.

## Introduction

Symbiotic interactions, in which long-term interactions take place between two partners belonging to different species, are common in nature [1]. These associations form a continuum ranging from parasitism to mutualism with respect to the outcome of the association (*i.e.* the cost or benefit for the host), and can be either facultative or obligate for the host. It has usually been assumed that parasitic, commensal or mutualistic symbionts interact in fundamentally different ways with their host. However, many bacterial symbionts can exist either as a mutualist or as a parasite, depending on their host [2],[3]. In addition, increasing reports in the literature indicate that the same molecular mechanisms are used by both parasitic and mutualistic symbionts to interact with their host [4]. If we focus on endocytobionts (*i.e.* symbionts living within the cells of their hosts), several mechanisms are known to be shared by parasites and mutualists, such as recognition and specific binding to the host cell, internalization within the cell, and finally intracellular survival and growth [5]. Common molecular mechanisms have been identified which are related to (i) communication processes, such as symbiosis/virulence factors that encode genes classically involved in secretion systems [6] (ii) survival and replication processes, including the expression of colonization factors [7], evading host immune systems [8], and regulating bacterial growth, and (iii) physiological processes involved in environmental adaptation, such as pH modification, induction of specific metabolic pathways, development of iron uptake strategies [9] and induction of stress proteins [10].

In this paper, we focus on *Wolbachia* (Rickettsiales), a well-known genus of bacteria that are reproductive parasites when associated with arthropod hosts [11] but mutualists when associated with nematodes [12]. Unlike other *Wolbachia* strains, which are generally facultative for their arthropods host, *Wolbachia* is necessary for oogenesis to be completed in the parasitoid wasp *Asobara tabida* (Hymenoptera, Braconidae) [13]. *Wolbachia* does not induce any cost or benefit affecting other life-history traits of *A. tabida* individuals, suggesting that the mutualistic interaction is exclusively due to the restoration of egg production in presence of *Wolbachia*. *A. tabida* is the only species within the genus *Asobara* to be dependent on *Wolbachia* for its oogenesis [14], which indicates this evolutionary transition towards obligatory dependence is recent, and makes it possible to investigate the molecular mechanisms underlying this dependence. A recent study has revealed that apoptosis is vastly greater in the nurse cells surrounding the oocytes in aposymbiotic ovaries than those in symbiotic ovaries [15]. Apoptotic processes are necessary for normal oogenesis to occur [16], but in the case of *A. tabida*, the absence of *Wolbachia* activates an early checkpoint during oogenesis [15]. This suggests that *Wolbachia* could be manipulating the host's apoptotic machinery, either indirectly or directly, as microbial pathogens often do to evade the host immune system [17]. A strategy usually associated with parasitism could thus be what has led to the swift transition from parasitism to obligate mutualism observed in this species.

In order to investigate the molecular mechanisms underlying host dependence, we used a comparative transcriptomic approach (Suppression Subtractive Hybridization: SSH) to compare symbiotic and aposymbiotic ovaries of *A. tabida* females. The heavy (HCH) and light (LCH) chains of ferritin, which form complexes involved in iron storage and the oxidation of labile iron in the cell [18], were found to be over-expressed in aposymbiotic individuals. This focus on ferritin is very relevant, due to the known major role played by iron in bacterial growth and infection [19], and hence its potential involvement in host-bacteria interactions. Furthermore, the anti-oxidant properties of ferritin have a direct effect in reducing global oxidative stress, and so could have an indirect effect on apoptotic processes. In addition, interactions between *Wolbachia* and iron metabolism are highly suspected. First, in filarial nematodes, all genes necessary for heme biosynthesis are retained in the reduce genome of *Wolbachia* strain *w*Bm [20] and almost absent in the genome of its host, the filarial nematode *Brugia malayi* [21]. Second, a recent study on *D. melanogaster* has also shown that *Wolbachia* infection could have a beneficial effect on fecundity by buffering iron fluctuations [22].

Starting from the observed over-expression of ferritin in aposymbiotic individuals, we focused on the influence of *Wolbachia* on host iron homeostasis, and the role of iron overload in apoptotic processes during oogenesis in *A. tabida*. We then extended our analysis to the facultative parasitic association known to exist between *Drosophila simulans* and *Wolbachia w*Ri and *Aegypti* cells lines stably infected by *Wolbachia w*Mel. We demonstrated that the influence of *Wolbachia* on iron homeostasis was not limited to *A. tabida*, and in fact constitutes a common mechanism used by both mutualistic and parasitic *Wolbachia*.

## Results

### Both heavy and light chains of ferritin were over-expressed in aposymbiotic individuals of *A. tabida* under standard conditions

**Figure 1:**
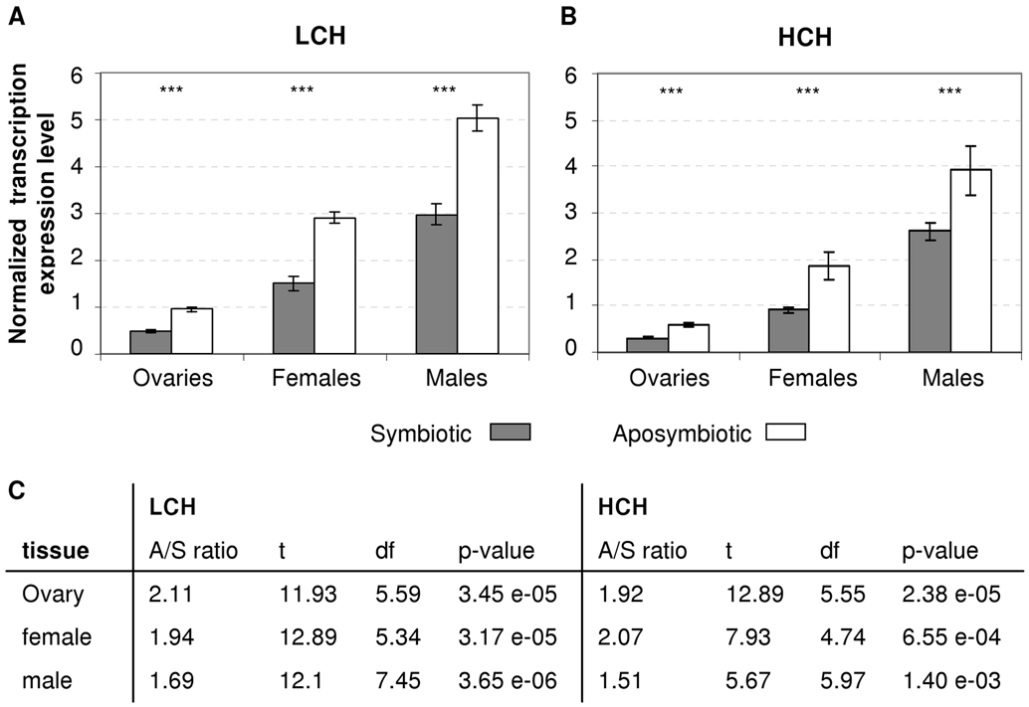
Ferritin expression in *A. tabida* under standard conditions. A–B. Expression of ferritin HCH (A.) or LCH (B.) relative to ribosomal L6 gene in aposymbiotic (white) and symbiotic (gray) ovaries, females and males. Asterisks indicate a statistically significant difference (t-test, ***: p<0.001) of ferritin expression in response to infection status. Expression was measured on 5 replicates of 10 individuals (Mean ratio +/−SE). C. Ratio between mean expression in aposymbiotic (A) and symbiotic (S) individuals, and details of statistical analysis (t-test with Welch correction on log-transformed data).

We performed a Suppression Subtractive Hybridization (SSH) between the distal part of symbiotic (S) and aposymbiotic (A) ovaries. Among all the potential candidates for involvement in apoptotic processes, we focused on ferritin, a protein complex involved in iron storage, and composed of HCH and LCH subunits. We first performed Real-Time quantitative PCR to confirm the over-expression of these genes, and showed that the heavy and light chains of ferritin were indeed both over-expressed in aposymbiotic *A. tabida* individuals (Fig. 1), suggesting that transcription of the two genes responded in similar ways to the absence of bacteria (see statistics in Fig. 1C). Ferritin over-expression was observed in the ovaries where the apoptotic phenotype was observed, in whole aposymbiotic females, but also slightly in males, even though no apoptotic phenotype was visible. These findings suggest that the over-expression of ferritin is a more global phenomenon controlled by the presence of *Wolbachia*, rather than being simply a response to the apoptotic phenotype observed in the ovaries.

### Genomic loci of host and *Wolbachia* ferritin

**Figure 2:**
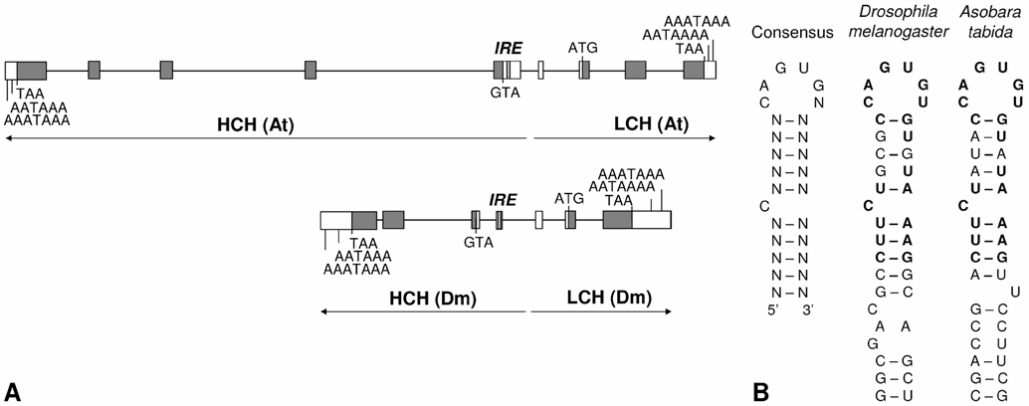
Genomic organization of Ferritin HCH and LCH. A. Genomic organization of ferritin in *A. tabida* (At) and in *D. melanogaster* (Dm) [48]. Introns are represented by lines, and exons by boxes to scale. Within exons, start and stop codons are indicated, delimiting the protein coding regions (shaded gray) and the untranslated regions (white). IRE is shown as dashed box. B. Sequence and secondary structure of the Iron Response Element (IRE) in *A. tabida* compared to the IRE consensus sequence [32] and the IRE of *D. melanogaster* [26].

As has already been shown for *Drosophila melanogaster*, *Anopheles gambiae*, *Apis mellifera* and *Bombyx mori* (review in [23]), ferritin HCH and LCH appeared to be clustered head-to-head in *A. tabida* to form a 9194-bp superlocus (Fig. 2A). In *Aedes aegypti*, LCH and HCH expression is regulated by cis-regulatory elements [24],[25]. Coordinated regulation has been demonstrated in several species, including *D. melanogaster* [26] and *A. aegypti* [27]. The ferritin intron/exon structure is known to be highly variable among insect species. Five and four exons were found in the HCH and LCH forms of *A. tabida* ferritin, respectively, without any evidence suggesting transposable element insertion or a repeated sequence. Multiple polyadenylation sites are present at the 3′-end of each gene, and could affect the length and stability of transcripts [28],[29]. HCH and LCH are 220-aa and 225-aa peptides, respectively, and possess a signal peptide for secretion with a cleavage site located between the 19^th^ and 20^th^ amino acid in the N-terminal region. *A. tabida* maintains all the ferroxidase residues of the ferritin HCH which are necessary for iron oxidation and iron absorption [30]. Finally, a canonical Iron Response Element (IRE), consisting of a hairpin hexaloop (5′-CAGUGN-3′) and a stalk disrupted by a bulge with an unpaired C nucleotide [31],[32], was found in the HCH transcript within the uncoding region of the first exon (Fig. 2B). This IRE could be responsible for translational control by iron via Iron Response Proteins (IRPs) [33]–[35].

We also characterized the 474-pb bacterioferritin gene (*bfr*) of the *Wolbachia* strain necessary for *A. tabida* oogenesis (strain *w*Atab3). It contained all the residues involved in ferroxidase activity [36], suggesting that the bacterioferritin is likely to be functional. This gene is highly conserved in the *Wolbachia* strains of the A-clade that have been sequenced (*w*Atab 1 or 2 of *A. tabida*, *w*Mel of *Drosophila melanogaster* [37], *w*Ri of *D. simulans* [38]). Indeed, only eleven variable sites have been detected within the entire nucleotide sequence of these strains. Seven out of them are specific to *w*Atab3, but six are synonymous substitutions. The only non-synonymous substitution (His *vs.* Tyr, position 79) has already been identified in *Wolbachia* strains from other clades (*w*Pip of *Culex quinquefasciatus* [39], *w*Bm of *Brugia malayi* [20]), and does not correspond to an amino acid in the ferroxidase center [36].

### *Wolbachia* limits parasite mortality after iron overload

**Figure 3:**
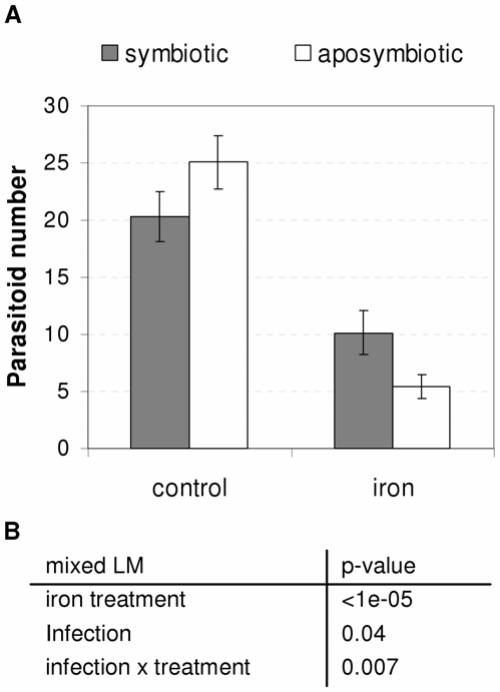
Influence of iron on wasp development. A. Emergence (mean±SE) of aposymbiotic (white) and symbiotic wasps (gray) following development on standard diet (control) or on iron-supplemented diet (iron). The results from two independent experiments (with 7 and 8 replicates for each condition, respectively) are pooled. B. The number of wasps emerged was analysed using mixed Linear Model (LM) with binomial error. The experiment was treated as a random factor, whereas iron treatment and infection status were treated as fixed factors.

In order to detect the potential effect of *Wolbachia* infection on host iron homeostasis, we artificially increased the iron load in the nutrient medium. Iron treatment was efficient since we measured a 5.24-fold increase of iron quantity in *Drosophila* larvae (t-test, t = 14.81, df = 4.13, p = 9.85e-05). The emergence of *A. tabida* adults slumped after a high iron load (Fig. 3, mixed LM, iron treatment: p<10^−5^), although iron overload had no effect on the development of *D. melanogaster* when they are not parasitized by *A. tabida* (mixed GLM with binomial error, iron treatment: p = 0.152). Furthermore, the decrease in wasp emergence was not attributable to a decrease in the infestation level (*i.e.* mean percentage of *Drosophila* hosts that have been parasitized±SE), which ranges from 84.1%±3.7 to 90.8%±3.9 for control treatment and from 86.2%±6.3 to 87.9%±5.0 for iron diet treatment, in symbiotic and aposymbiotic wasps, respectively. These findings suggest that the parasitoid development was affected by the iron increase, probably because parasitoids usually develop in the highly buffered environment of *Drosophila* larvae. Interestingly, while symbiotic individuals still suffer from higher iron load, the deleterious effect on wasp development was less pronounced than in aposymbiotic individuals (2.00-fold *vs.* 4.64-fold reduction; Mixed LM, iron treatment×infection, p = 0.007). This interaction between iron load and bacterial infection suggests that the presence of *Wolbachia* could act as a buffer in the wasp iron homeostasis after iron overload, and hence limit the deleterious effect of labile iron.

### Little iron is absorbed by the wasps, and ferritin expression does not increase after iron treatment

In order to find out whether iron uptake differed between symbiotic and aposymbiotic wasps, we measured the iron absorbed by the wasp. We detected a marginally significant increase in iron content after the administration of an iron diet (ANOVA, F_1,14_ = 3.70, p = 0.07), which ranged from 27.39±0.95 (mean±SE in nmol of iron/mg of protein) to 29.34±1.04 for symbiotic females, and from 27.99±1.24 to 29.92±0.50 for aposymbiotic ones. However, we did not detect any effect of *Wolbachia*'s presence on the total iron absorption nor any interaction between *Wolbachia* and iron treatment (ANOVA, infection status, F_1,14_ = 0.31, p = 0.58; iron treatment × infection, F_1,14_ = 0.004, p = 0.95).

Ferritin expression was determined after iron treatment in both symbiotic and aposymbiotic females. We did not detect any over-expression of host ferritin or bacterioferritin transcripts in response to iron treatment (Table 1). This absence of a transcriptional response was not attributable to any change in bacterial density in response to iron load ([WSP]/[18S]×10^5^ = 7.36±0.61 (mean±SE) for control treatment, and 7.83±1.13 for iron treatment; t-test on log-transformed data: t = −0.44, df = 5.49, p = 0.68). One interpretation is that emergence of parasitoids after iron overload occurred only in *Drosophila* larvae with efficient iron homeostasis and low iron content in their body, or in those larvae that were more efficient iron consumers. Alternatively, we could be observing a variation in the wasp's tolerance to iron. However, this hypothesis is not well supported by the fact that the increase in iron content in the wasp was very limited after the iron treatment compared to that in the *Drosophila* larvae, and also that no transcriptomic response was detected.

**Table 1:** Ferritin expression after iron treatment in *A. tabida*.

**A**							
**Experimental condition**	**LCH**		**HCH**		**Bacterioferritin**		
	**mean**	**SE**	**mean**	**SE**	**mean**	**SE**	
Symbiotic/control	1.06	0.05	0.24	0.01	11.31 e-04	1.66 e-05	
Symbiotic/iron	1.13	0.05	0.25	0.01	11.07 e-04	1.83 e-05	
Aposymbiotic/control	2.89	0.15	0.64	0.05	-	-	
Aposymbiotic/iron	2.73	0.14	0.62	0.03	-	-	
**B**							
**ANOVA on log-transformed data**	**LCH**		**HCH**		**Bacterioferritin (t-test)**		
	**F(1,16)**	**p-value**	**F(1,16)**	**p-value**	**T**	**df**	**p-value**
Iron treatment	8.68 e-06	0.99	0.01	0.92	−0.06	7.8	0.95
Infection	352.00	8.48 e-13	311.60	2.27 e-12	-	-	-
Infection × treatment		NS		NS	-	-	-

**A.** Relative expression of Ferritin HCH, LCH and of bacterioferritin in response to iron treatment and infection. Expression was measured on 5 replicates of 10 females. **B.** ANOVA and t-test on log-transformed data.

### Apoptosis is induced in the ovaries of *A. tabida* after iron overload

**Figure 4:**
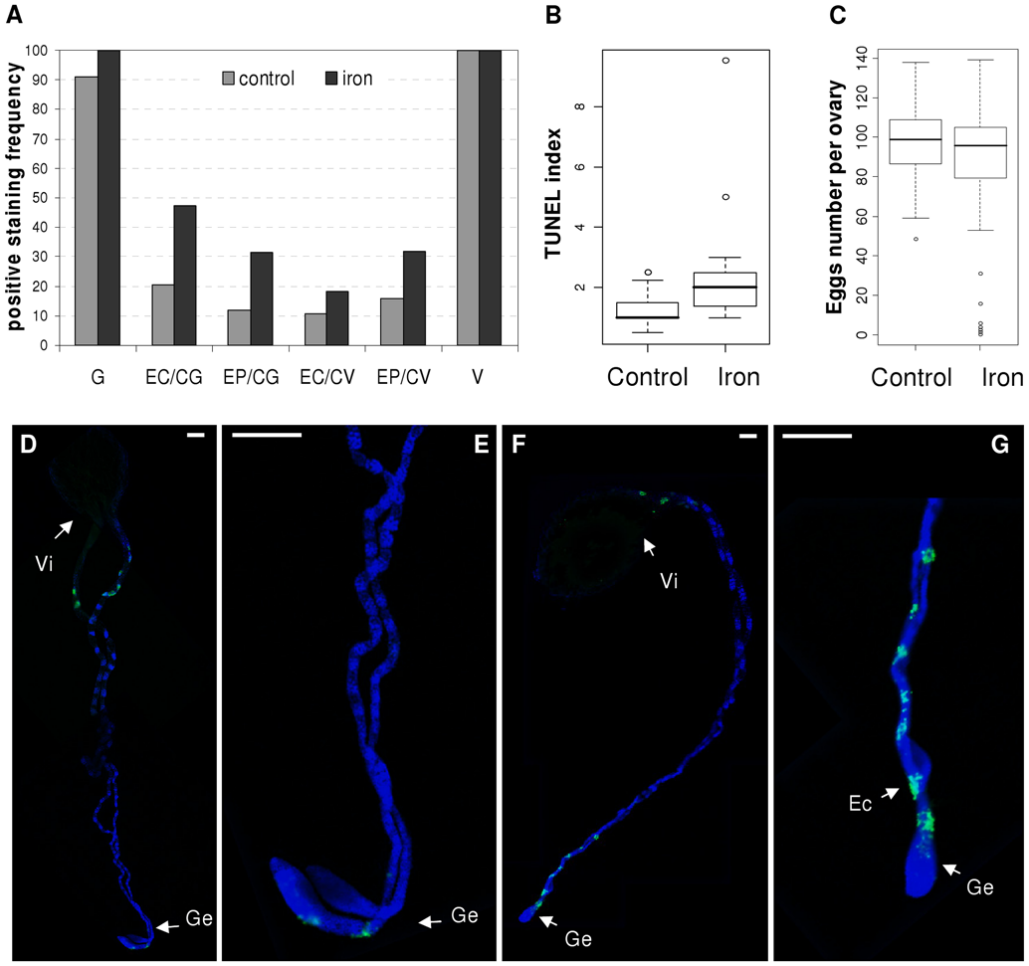
Ovarian phenotype in response to iron stress. A. Distribution of TUNEL staining (whole Egg Chamber (EC) or Erratic Points (EP)) in the Germarium (G), Close to the Germarium (CG), Close to the Vitellarium (CV), or in the Vitellarium (V). B. TUNEL index of ovaries from females reared under standard (control, n = 17) or iron-supplemented diets (iron, n = 19). C. Oocyte load of females developed under standard (control, n = 60) or iron-supplemented diets (iron, n = 60). Box-and-whisker plot shows the extreme of the lower whisker, the lower hinge, the median, the upper hinge and the extreme of the upper whisker for each group. D–G. TUNEL (green) and DAPI (blue) staining of ovaries. (D, E) PCD occurred only in the germarium (Ge) and during the dumping near the vitellarium (Vi) under control conditions. (F, G) PCD is induced in egg chambers (Ec) close to the germarium after iron-supplementation. Scale bar: 100 µm.

In *Drosophila*, stress induces several different apoptotic checkpoints that control egg production [16]. In *A. tabida*, there was a marginally significant reduction in oocyte load after iron treatment (Wilcoxon, W = 1484, p = 0.07, Fig. 4C), with some ovaries being totally devoid of eggs after iron overload. These empty ovaries were similar to those observed in ovaries of aposymbiotic females where apoptosis was detected early during the oogenetic process [15]. In response to iron treatment, TUNEL staining was enhanced in ovaries from symbiotic females (Fig. 4), indicating an increase in apoptosis (Wilcoxon, W = 241, p = 0.01). This increase in TUNEL staining was particularly marked in egg chambers close to the germarium, and was detected either in all the nurse cells of an entire egg chamber, or in only some of them (erratic points). Thus, a toxic effect of iron on oogenesis was detected despite the limited impact of iron diet treatment on iron concentration and ferritin expression in *A. tabida*, which reinforces the idea that *A. tabida* is very susceptible to iron.

### *Wolbachia* influences iron absorption and ferritin expression in *Drosophila simulans* after iron overload

In order to find out whether the manipulation of iron homeostasis by *Wolbachia* also occurs in other species, we manipulated the iron content in the diet of the facultative association between *D. simulans* and its *Wolbachia w*Ri.

**Figure 5:**
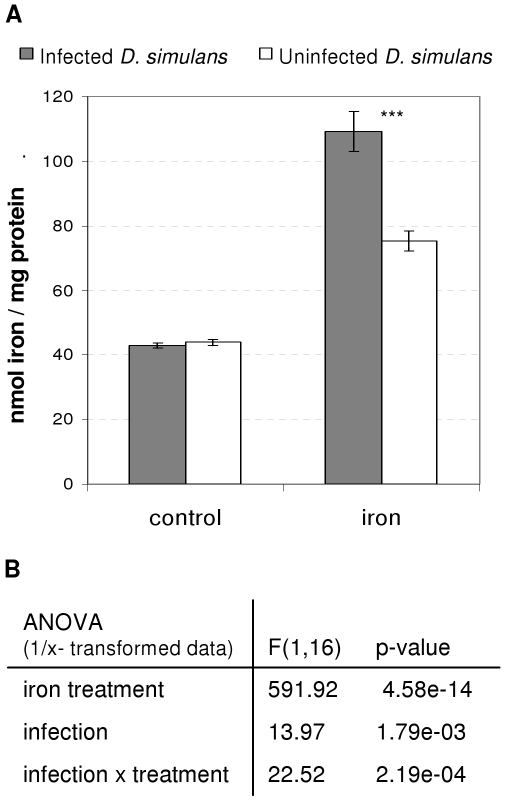
Total iron absorption by *D. simulans*. A. Mean iron absorption (ng iron/mg protein±SE) in infected and uninfected *D. simulans*, after standard (control) or iron-supplemented treatment (iron). Measurements were performed on 4 replicates of 10 females per treatment. Asterisks indicate a statistically significant difference (t-test on 1/x-transformed data with TukeyHSD adjustment, ***: p<0.001) B. Details of statistical analysis (ANOVA on 1/x-transformed data).

Although *Wolbachia* did not have any effect on iron absorption under control conditions, we found that *Wolbachia* had a major impact on total iron absorption under iron-supplemented conditions (Fig. 5, statistics in Fig. 5B). Infected flies did indeed absorb more iron than uninfected ones after iron treatment (TukeyHSD, df = 6, p adj = 2.33e-5). However, it was not technically possible to differentiate between the iron absorbed by the fly and that absorbed by *Wolbachia*.

**Figure 6:**
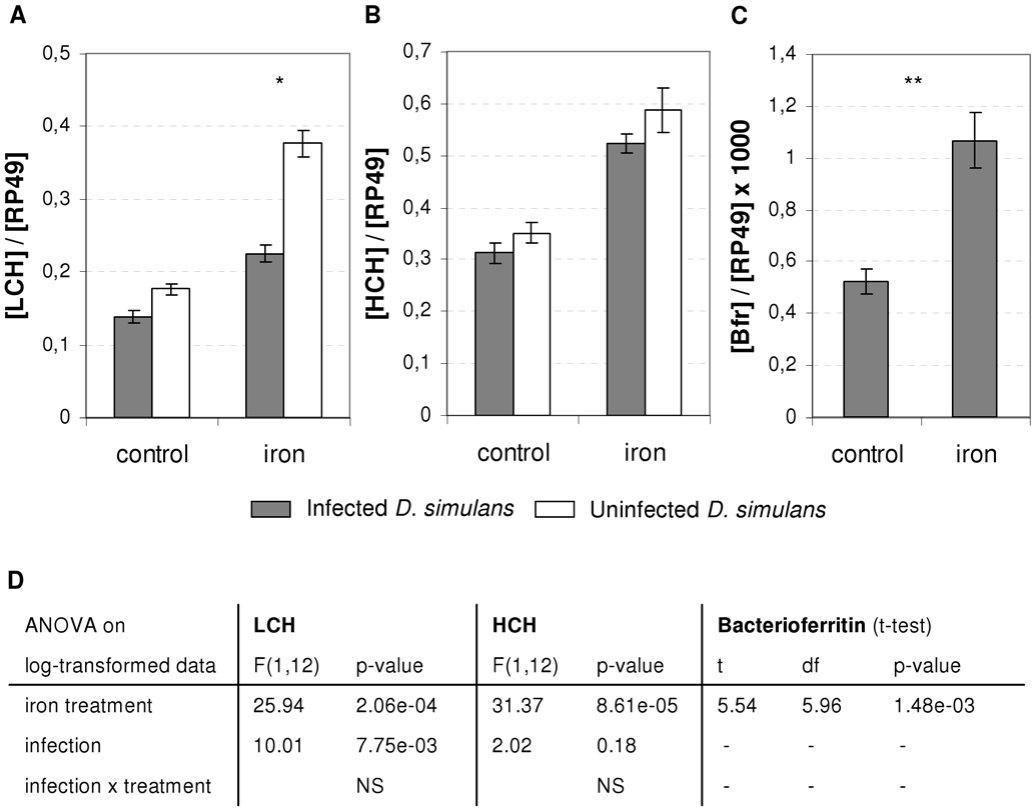
Ferritin expression in *D. simulans*. A–C. Expression of ferritin LCH (A), HCH (B) or bacterioferritin (C) relative to ribosomal gene RP49 in aposymbiotic females (white) and symbiotic females (gray) in response to iron treatment. Expression was measured on 4 replicates of 5 females (Mean ratio±SE). Asterisks indicate a statistically significant difference (t-test with TukeyHSD adjustment, **: p<0.01, *: p<0.05) in gene expression in response to infection status for LCH or iron treatment for Bfr. D. Details of statistical analysis (ANOVA and t-test with Welch correction on log-transformed data).

We subsequently looked at ferritin expression in response to iron treatment and infection (Fig. 6, statistics in Fig. 6D). As for iron absorption, *Wolbachia* had no effect on ferritin expression under control conditions. In response to iron treatment, HCH expression increased 1.67 times, but there was no interaction with infection. LCH expression also increased after iron treatment, but this increase was lower in infected (1.63-fold increase) than in uninfected females (2.13-fold increase). This could be explained by the transcriptional response of bacterioferritin, the expression of which doubled after iron treatment (Fig. 6C, statistics in Fig. 6D). This increase in bacterioferritin expression cannot have been attributable to bacterial density as we did not observe any change in response to iron treatment ([WSP]/[RP49] = 1.07±0.09 (mean±SE) for control treatment, and 0.93±0.11 for iron diet treatment; t-test on log-transformed data : t = 0.76, df = 18.68, p = 0.45).

Finally, we did not detect any influence of *Wolbachia's* presence on morphometric traits (wing size) or offspring production under control or iron diet treatment conditions (data not shown). This suggests that *D. simulans* can easily adapt its metabolism to the change in iron homeostasis observed after iron overload and *Wolbachia* infection. The longevity of the infected flies was greater than that of the uninfected ones under both control and iron diet treatment conditions (Fig. S1), suggesting that *Wolbachia* infection confers some benefit. However, the effect of iron treatment was not significant, and there was no interaction between infection and iron diet (Cox model, infection status: p = 0.01, iron treatment: p = 0.14, infection × iron treatment: p = 0.50).

### *Wolbachia* limits the increase in ferritin expression after iron overload in *Aedes aegypti* cells

**Figure 7:**
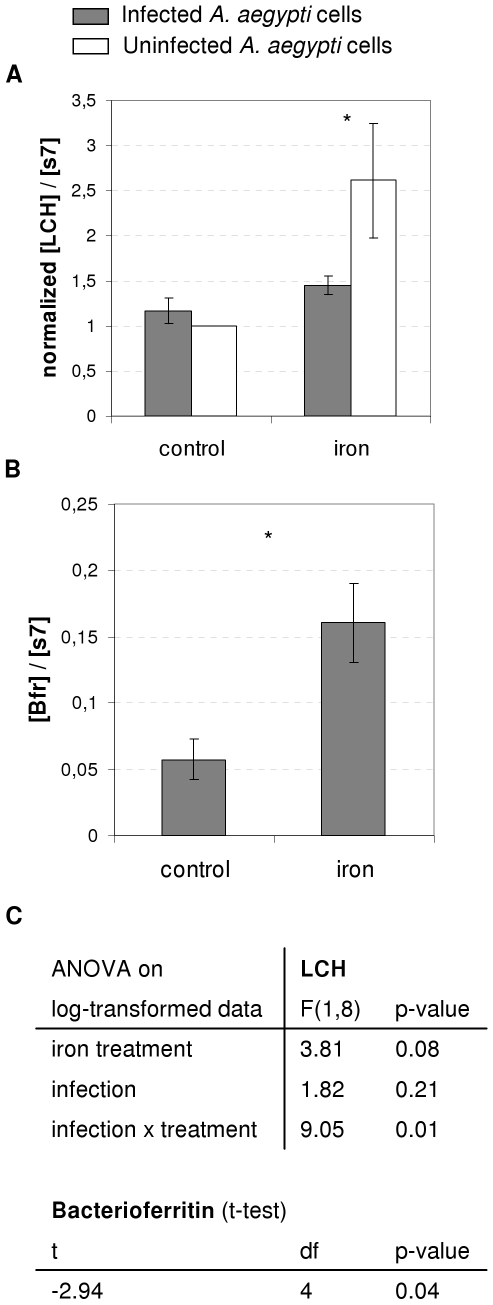
Ferritin expression in *A. aegypti* cells. A–B. Expression of ferritin LCH (A) and bacterioferritin (B) relative to ribosomal gene S7 in uninfected (white) and infected cells (gray), in response to iron treatment. LCH expression values were normalized by the mean ratio ([LCH]/[S7]) of uninfected cells in the absence of iron. Expression was measured on 3 biological replicates (Mean ratio±SE). Asterisks indicate a significant statistical difference (t-test with TukeyHSD adjustment, *: p<0.05) in gene expression in response to infection status for LCH or iron treatment for Bfr. C. Details of statistical analysis (ANOVA and t-test on log-transformed data).

We then looked at the association between *A. aegypti* cells and *Wolbachia w*Mel. Growing conditions could easily be manipulated by adding iron, thus directly affecting host cell metabolism. Ferritin expression in infected and uninfected *A. aegypti* cells was not significantly different under standard culture conditions (tukeyHSD, df = 4, p adj = 0.66, Fig. 7A). When iron was added, the LCH ferritin level remained constant in RML12 cells infected by *w*Mel (referred as RML12-wMel, TukeyHSD, df = 4, p adj = 0.87), whereas it increased significantly in uninfected cells (TukeyHSD, df = 4, p adj = 0.05). This enhanced LCH ferritin level was more than two and a half times higher (TukeyHSD, df = 4, p adj = 0.03) than that of the control (*i.e.* cells grown without iron). As the global statistical analysis revealed a positive interaction between iron and infection (Fig. 7C), we hypothesized that the *Wolbachia* bacterioferritin could be compensating for the reduced expression of host ferritin under iron-overload conditions. To confirm this hypothesis, we estimated the difference between the relative levels of Bfr expression in infected cells grown in medium with and without the iron supplement. Bfr expression was three times higher in iron-supplemented than in standard medium (Fig. 7B, statistics Fig. 7C). The lack of ferritin regulation in infected individuals in response to iron overload indicates that the symbiotic bacterium *Wolbachia* reduces the need of host cells to up-regulate ferritin upon iron exposure.

## Discussion

### Role of iron metabolism in host-parasite interactions

Iron is essential for survival in all organisms, since it is used as a cofactor or catalyst in electron transport. Bacteria also need iron to grow and to maximize the infection process [40],[41]. Hence, competition for iron can emerge between partners in host-bacteria associations. Many proteins scavenge iron, including transferrin, lactoferrin, ferritin or siderophores in eukaryotes, and bacterioferritin or siderophores in prokaryotes.

Although iron is essential for most organism, an excess of iron in the cell is harmful. Indeed, labile iron catalyses Reactive Oxygen Species (ROS) via the Fenton reaction in the presence of H_2_O_2,_ and activates the immune response and apoptosis [42]. Ferritin is an iron storage protein, which can complex labile iron and buffer the intracellular labile iron pool. Because its expression is regulated by iron [43] and by oxidant residues via an Antioxidant Responsive Element (ARE) [44],[45], ferritin contributes to iron homeostasis and oxidative stress reduction [18].

Finally, iron is involved in the modulation of innate immunity mechanisms against pathogenic bacteria in mammals, such as the production of ROS and RNI (Reactive Nitrogen Intermediates), and limiting bacterial access to iron can also lower their infectivity [46]. In addition, HCH ferritin expression is under the control of the NF-κB signaling pathway in mammals. This pathway plays a major role in the immune response, inducing the expression of immune-related genes, and limiting ROS-induced apoptosis [47]. In *D. melanogaster*, ferritin genes possess two NF-κB binding domains [48], and ferritin up-regulation has also been described after immune challenge in starfish [49], the horseshoe crab [50] and A*mphioxus* [51].

### *Wolbachia* influences iron metabolism in various biological systems

Our results strongly suggest that iron plays a pivotal role in the interaction between *Wolbachia* and its hosts. We detected a global influence of *Wolbachia* on ferritin expression in *A. tabida* under standard conditions, but also in *D. simulans* and *A. aegypti* cells after iron supplementation. The fact that similar results have been obtained using different systems suggests that the effect observed in *A. tabida* is not due to a direct action of the antibiotic treatment, but rather to an effect of the absence of *Wolbachia*. In *D. simulans* and the *Aedes* cell lines, we also observed a transcriptional response by the *Wolbachia* itself, with bacterioferritin expression increasing in response to an iron-supplemented diet. Possible interactions between *Wolbachia* and iron metabolism have already been suggested, since almost all the genes necessary for heme biosynthesis and also bacterioferritin, are retained by the *Wolbachia* infecting the nematode *Brugia malayi* [20], *Drosophila melanogaster* [37] and *Culex quinquefasciatus* [39]. Different lines of evidence suggest that this pathway could contribute to the *Wolbachia*-nematode mutualism [20]. In particular, several nematodes, among which *B. malayi*, have lost the machinery for heme production and depend on external sources [52]. *Wolbachia* infection in filarial nematodes could allow for this provision and explain why *Wolbachia* is a mutualist in these organisms while it is a parasite in others. However, this does not preclude any interaction between iron metabolism and *Wolbachia* in other systems. For example, *Wolbachia* participation in iron homeostasis has been shown to be beneficial in *D. melanogaster* under conditions of nutritional stress [22]. Our data provide clear evidence that *Wolbachia* does indeed interact with iron metabolism at the molecular level in both the host and the bacterium. This means that the intracellular life-style of *Wolbachia* may rely on similar pathways in mutualistic or parasitic associations, which confirms that iron plays a major role in host-symbiont interactions [46].

Since the primary function of ferritin in insects is to capture, store and transport iron [53],[54], the decrease in host ferritin mediated by *Wolbachia* infection could be explained by two distinct mechanisms: (i) *Wolbachia* could repress the expression of host ferritin, in order to have more easily access to the iron within the cell, or (ii) *Wolbachia* could scavenge iron in the cell, thus reducing the concentration of labile iron, and leading to reduced expression of ferritin. This last mechanism is supported by the results obtained for the association between *D. simulans* and *Wolbachia w*Ri, where the iron uptake following an iron overload was greater in infected females than in uninfected ones. This difference in iron uptake could be due to the iron scavenged by *Wolbachia*, as suggested by the increase in bacterioferritin expression after iron-supplementation. Similar results were also observed in *A. aegypti* cells, where the absence of transcriptional response of the light chain in infected cells could be offset by an increase in the expression of bacterioferritin.

### Influence of endosymbionts on host functions: from cellular physiology to immune system evasion?

Intracellular infection by bacteria may alter the cellular environment. This alteration can be a side effect of infection, a defense reaction of the host, or a manipulation induced by the symbiont to allow it to survive within the host cell. For example, the presence of an intracellular bacterium in an organism is generally detected by the host, which can in turn induce stress in the infected cell (production of toxins, ROS …), activate the immune system, or enter into apoptosis [55]. In order to allow them to persist within the host cells, micro-organisms have developed various strategies to counter host defense strategies, including producing anti-oxidant bacterial molecules, manipulating the expression of host genes, blocking immune effectors, and activating/inhibiting apoptosis [56],[57].

It was shown recently that *Wolbachia* induces stress (ROS production) when present in Aa23 *Aedes albopictus* cell cultures [58]. 2D-PAGE analysis has shown that *Wolbachia* infection up-regulates various host anti-oxidant proteins in cell cultures, and that *Wolbachia* bacterioferritin and Fe superoxide dismutase were highly expressed [58]. Our results confirm the expression of bacterioferritin in *Aedes* cell cultures, and also in whole insects. In addition, we have also demonstrated a transcriptional response of this gene to environmental conditions in *D. simulans* and *Aedes* cell lines. In both biological systems, there was no increase in bacterial densities after iron overload, suggesting that iron is not a limiting factor for bacterial growth. This could indicate that overexpression of bacterioferritin is related to ROS limitation rather than being a metabolic requirement. This could also explain why, in these two systems, an effect of bacterial infection was only detected in iron supplemented medium. Together with the results reported by [58], this suggests that host and symbiont share the task of limiting the cytotoxic effects resulting from infection and from environmental conditions.

A striking finding is that the presence of *Wolbachia* in host cells reduces ferritin expression, while it has generally been shown to be up-regulated by infection, even in close relatives of *Wolbachia*, such as *Anaplasma phagocytophilum* in human neutrophils [59] or *Rickettsia montanensis* in ticks [60]. This finding might be related to the fact that the only Rickettsiales in which bacterioferritin has been found is *Wolbachia*. These bacteria could rely on other systems to limit oxidative stress and its consequences, such as apoptosis, as has been demonstrated for the human pathogens *Rickettsia rickettsii* (*Rickettsiaceae*) and *Anaplasma phagocytophilum* (*Anaplasmataceae*) [61],[62].

### Is iron homeostasis involved in the evolution of dependence in *A. tabida*?

Following iron overload, we did not detect any effect of infection status on fly development and life-history traits (size, longevity and oogenesis) in the facultative association between *D. simulans* and *w*Ri. This contrasts with the fecundity benefits observed in *D. melanogaster* [22], and could be due to differences in bacterial density or host tissue localization between the *w*Ri and *w*Mel strains [63]. We also found that *Wolbachia* infection had an overall protective effect on developmental success in *A. tabida*, which could be due to the ability of *Wolbachia* to scavenge and thus to buffer the iron present within the cell. The high developmental mortality in iron-fed wasps suggests that *A. tabida* has a very limited plasticity to changes in iron concentration. This difference between *A. tabida* and *Drosophila* species is probably related to their differing lifestyles: development in a highly buffered environment, consisting of *Drosophila* larvae for the former, *vs.* heterogeneous substrates for the latter.

We also found that the toxicity of iron in infected *A. tabida* females led to an increase in apoptosis in the ovaries comparable to the phenotype observed in aposymbiotic females in the most extreme cases observed. This result reinforces the idea that *A. tabida* is very susceptible to iron, since this effect is observed despite a moderate increase of iron concentration in the wasps. This also raises the possibility that iron metabolism may be linked to the evolution of dependence. Indeed, egg production is controlled in *Drosophila* by the induction of two main apoptotic checkpoints in reaction to deleterious external stimuli, such as nutrient deprivation, cytotoxic chemicals and abnormal development [16]. If *Wolbachia* contributes to limiting ROS in *A. tabida*, then removing the bacteria or rearing the wasps on iron supplemented-medium could generate the accumulation of ROS during oogenesis, and the further activation of checkpoints. According to this hypothesis, removing *Wolbachia* would lead to ROS accumulation and the over-expression of host anti-oxidant genes, including ferritin. However, the low plasticity of *A. tabida* could lead to a response that is insufficient to counteract the apoptotic process in aposymbiotic ovaries. To comfort the link between iron metabolism and wasp dependence on *Wolbachia*, developing wasps on low-iron diet would be required to determine whether this affects oxidative stress and apoptotic processes. Unfortunately, all our attempts to chelate iron by adding BPS (Bathophenanthroline disulfonic acid) in the *Drosophila* food did not reduce the iron content in the wasp and it was thus not possible to limit iron in this *Drosophila*/parasitoid system.

Taken together, these data suggest that the presence of *Wolbachia* could dramatically affect the cellular physiology of its hosts. Consequences of endosymbiont infection may thus extend far beyond their effect on reproduction, which widen their possible impact on the evolution of their hosts. Compensation or tolerance may easily evolve for highly prevalent symbionts such as *Wolbachia* and contribute in some extreme cases to the emergence of dependence in an initially parasitic association.

## Materials and Methods

### Biological systems

#### *Asobara tabida*

*Asobara tabida* (Hymenoptera: Braconidae) is a solitary parasitoid species, and is naturally infected by three strains of the intracellular bacterium *Wolbachia* (*w*Atab1, *w*Atab2 and *w*Atab3) [64]. *w*Atab1 and *w*Atab2 induce cytoplasmic incompatibility, and only *w*Atab3 is required for oogenesis completion [65]. *A. tabida* females lay eggs into the first or second instar larvae of *Drosophila*. After *Drosophila* pupation, the parasitoid becomes an ectoparasite, and consumes its host before pupating and emerging. Wasps were maintained under controlled rearing conditions (20°C, 12 light/dark (LD) cycle) on axenic medium.

A parasitoid wasp strain from Pierrefeu (France) has been maintained by regular sib-matings on uninfected *Drosophila melanogaster*. The symbiotic strain used in this study was a derived Pierrefeu strain obtained by moderate antibiotic treatment [65], and it contains only the obligatory *Wolbachia* strain *w*Atab3. The line used is stable, and has been maintained without antibiotic treatment for about 100 generations.

Aposymbiotic females are sterile, which makes it impossible to establish and maintain aposymbiotic lines, and so antibiotic treatments were administered just before the experiment in order to obtain aposymbiotic wasps [13].

#### *Drosophila simulans*

*Drosophila simulans* (Diptera: Drosophilidae) was trapped in Antibes (France), and was naturally infected by the *w*Ri strain. Aposymbiotic flies were produced by two successive antibiotic treatments [13], followed by 12 generations without antibiotic treatment. Flies were maintained under controlled rearing conditions (20°C, 12 LD cycle) on axenic medium.

#### *Aedes aegypti* RML12 cell line

The *Aedes aegypti* RML12 cell line derived from larvae [66] and infected by *Wolbachia* strain *w*Mel was kindly provided by Prof. Scott O'Neill (University of Queensland, Australia). A subline devoid of *Wolbachia* was obtained by tetracycline treatment as described elsewhere [67]. Infected and uninfected RML12 cell lines were grown at 26°C in 25 cm^2^ culture flasks (Greiner Bio-One, Germany) containing equal volumes of Mitsuhashi/Maramorosh (Bioconcept, Switzerland) and Schneider' insect medium (Sigma, France), supplemented with 10% (vol/vol) heat-inactivated fetal bovine serum and penicillin/streptomycin (50 U/50 µg per ml) (Gibco, Invitrogen, France).

### Characterization of the ferritin genes

#### Insect ferritin

*A. tabida* ferritin ESTs obtained by Suppression Subtractive Hybridization were full-length sequenced using the GeneRacer Kit (Invitrogen, France) in accordance with the manufacturer's instructions. The genomic region was sequenced (EuroBlue Taq, Eurobio, France) to check the clustering of Light- and Heavy-chains at the same genomic locus, and the potential presence of Iron Response Elements (IRE) in introns. Signal peptides were detected using SignalP-3.0 software [68]. Genomic ferritin sequence has been deposited in the EMBL database under accession number FN395057.

#### Bacterioferritin (Bfr)

Whole bacterioferritin CDS was amplified using consensus primers (Bfr-F1: ATG AAT GAA GAG ATA GTA and Bfr-R1: TAT TTG TGT TCT TAA ATA) in *A. tabida* (*w*Atab3 and one of the two other strains (*w*Atab1 or *w*Atab2)) and in *D. simulans* (*w*Ri). The bacterioferritin sequences have been deposited in the EMBL database under accession numbers FN395058 to FN395060.

### Quantitative expression by Real-Time RT-PCR

#### *A. tabida*

We used a Real-Time quantitative RT-PCR technique to measure ferritin expression. Young individuals (0–2 days old) were isolated from symbiotic and aposymbiotic lines (5 replicates of 10 females, 10 males, or 10 ovaries (only the distal part which does not contain eggs was dissected in a drop of A-buffer ([KCl] = 25 mM, [MgCl_2_] = 10 mM, [Sucrose] = 250 mM, [Tris] = 35 mM, pH = 7.5)). Total RNA was extracted as described in [69]. To eliminate any contamination by DNA, samples were treated with DNase (TURBO DNA-free, Ambion, Applied Biosystems, TX, USA). First-strand cDNA was synthesized with SuperScript III (Invitrogen, France) in accordance with manufacturer's instructions, starting from 500 ng of total RNA, and using oligodT primers (or random primers for Bfr). The following primers were used for quantitative PCR: HCH-At-3F: CCC ATG GGT GAT CAT CAT TT and HCH-At-2R: CCA GTC ACC AGA TCC CAA GA (amplicon: 233 bp); LCH-At-F: TAC CAA GAG AGG CGG AAG AA and LCH-At-R: CGA GGA ACT CCT CCT CAA TG (amplicon: 218 bp); Bfr-F2: TGC GAT TAT CTT TTG CAT and Bfr-R2: TCG TTT CTT TGA TAT CCT (amplicon: 245 bp). Quantitative RT-PCR was performed using LightCycler LC480 system (Roche, France) as follows: 5 min at 95°C, 35 times [15 sec at 95°C, 10 s at 58°C (or 54°C for Bfr), 20 sec at 72°C], 20 sec at 70°C. The reaction mixture consisted of 0.5 µM of each primer, 5 µl of Fast SYBR-Green Master Mix (Roche, France), and 4 µl of diluted cDNA (corresponding to 25 ng of cDNA). Standard curves were plotted using 7 dilutions (10 to 10^7^ copies) of a previously amplified PCR product purified using Nucleospin Extract II kit (Machery-Nagel, France). The number of gene copies was calculated as described in [70]. The L6-ribosomal gene (RibL6-At-F: CAC CGA TGA TGA GCT TGT CT and RibL6-At-R CCC GAG AAT CTT AAC GAT GA, amplicon: 154 bp) was used as housekeeping gene for quantitative analyses. This gene was not differentially expressed in response to infection and iron treatment. In addition, similar results were obtained when Elongation factor 1γ and β-tubulin were used to normalize expression (data not shown).

#### *D. simulans*

The same procedure was used to quantify the ferritin in *D. simulans* (4 replicates from 5 whole females), except that the total RNA of the *D. simulans* females was extracted using RNeasy kit (Invitrogen, France). The following primers were used for quantitative PCR: HCH-Ds-F: GAA TTT TGC TGT GCT GAT TT and HCH-Ds-R: CCC TGT AAG GAG GTG GAG AT (amplicon: 148 bp); LCH-Ds-F: CGT CTA CCT GTT CGA CGA GT and LCH-Ds-R: GTG GCT GCT TTG ATA ATG CT (amplicon: 190 bp). The RP49 gene [71] was used as housekeeping gene for quantitative analyses.

#### *A. aegypti* cell line

For total mRNA extraction, three biological replications were done using 5×10^7^ cells of each line. Cells were homogenized in trizol solution, and total RNA was extracted using chloroform/trizol method as recommended. The total RNA contained in the aqueous phase was precipitated by 2 M LiCl, overnight at −20°C. After centrifuging at 16'000 g for 20 min, pelleted RNA was washed twice with cold ethanol (70%), then dried by vacuum centrifugation, and dissolved in 30 µl of DNase/RNase-free water. The extracted RNA was treated with DNase (TURBO DNA-free, Ambion, Applied Biosystems, TX, USA), in accordance with the manufacturer's recommendations. RNA was reverse transcribed using SuperScript III and random primers (Invitrogen, France), in accordance with manufacturer's instructions. For quantitative PCR, cDNAs were diluted to 10 ng/µl, and the following primers were used: cell ferritin (LCH2eF: CTC AAA GGC GGA GTT ATT GG and LCH3eR-1: ATC GTA CGT GGC CTT GTT GT, amplicon: 183 bp); bacterioferritin (see above); host ribosomal gene 7S rRNA [72]. Quantitative PCR was performed using LightCycler LC480 system (Roche, France) as follows: 10 min at 95°C, 35 times [15 sec at 95°C, 10 s at 57°C (or 54°C for Bfr), 20 sec at 72°C], 20 sec at 70°C. The reaction mixture consisted of 0.4 µM of each primer, 10 µl of Fast SYBR-Green Master Mix (Roche, France), and 2 µl of diluted cDNA (corresponding to 20 ng of cDNA) in a total reaction volume of 20 µl.

### Exposure to iron and life-history traits

#### Iron treatment

Seventy *D. melanogaster* eggs were placed on standard diet (Control), or on standard diet supplemented by FAC (Ferric Ammonium Citrate, Aldrich, MO, USA) 20 mM (Iron). The diet was kept unchanged all along larval development. Three *A. tabida* females were allowed to lay eggs on the larvae hatched from the eggs. As parasitoid larvae feed on the hemolymph of their *Drosophila* host, iron absorbed by the fly is available for the developing wasp.

For *D. simulans*, seventy eggs were placed on standard diet (Control) or on standard diet supplemented by FAC 20 mM (Iron).

In all the cell cultures, the medium was replaced by a fresh medium without fetal bovine serum when 80% confluence had been reached, and the cultures were then incubated overnight (∼18 hours) [73]. After the incubation period, fresh medium supplemented with FAC 100 µM was added, and incubation was continued for a further 18 hours. For control experiments, the infected and uninfected cell lines were treated as above, but the iron was omitted. Cells were collected by centrifuging; and Trypan Blue staining was used to estimate the proportions of live/dead cells. In all experiments, including the controls, cell viability was around 96% (mean) and ranges from 92% to 99%. Pelleted cells were quickly frozen in liquid nitrogen, and stored at −80°C until used.

#### Development of *A. tabida*

Seventy *Drosophila* eggs were introduced in each vial, and three *A. tabida* females were introduced for parasitization. Four different treatments were performed: development on standard medium, on standard medium plus antibiotic, on iron-supplemented medium and on iron-supplemented medium plus antibiotic. The experiment was repeated two times with 7 and 8 replicates for each treatment, respectively. As a control, the same protocol was performed without adding the wasps (n = 8 replicates per condition). The *Drosophila* and *A. tabida* that emerged were counted.

#### Total iron absorption

Iron was measured using a Ferrozine assay adapted from [74] with 15 *A. tabida* females, 10 *Drosophila* females or 10 *Drosophila* larvae (stage L3), except that (i) *Drosophila* heads were removed in order to avoid any interference due to their red eye coloration and (ii) purple coloration was measured at 562 nm, and the background at 750 nm was deduced (Xenius spectrophotometer, Safas, Monaco).

#### Bacterial density

DNA from 6 females was individually extracted using DNeasy kit (Qiagen, France) in accordance with manufacturer's instructions. Quantitative PCR was performed as described below, using 81-F: TGG TCC AAT AAG TGA TGA AGA AAC/691-R: AAA AAT TAA ACG CTA CTC CA primers (Tm: 52°C) to quantify total *Wolbachia* density, and 18S.lo1/NS58+2 [75] or RP49 F/R [71] as a normalizer for *A. tabida* and *D. simulans*, respectively.

#### Ovarian phenotype

Ovaries of *A. tabida* females reared under control or iron-supplemented diets were dissected in PBS, and individually transferred onto a slide using Roti-Liquid Barrier (Roth, France). Ovaries were either mounted in PBS to count the eggs (n = 60 per treatment), or stained as follows for the TUNEL assay (Terminal deoxynucleotidyl transferase-mediated dUTP- biotin Nick End Labeling, n = 17 and 19 for control and iron, respectively). Ovaries were fixed in paraformadehyde 4%-PBST 0.1% for 30 min, washed with PBS, made permeable with 20 µg/mL Proteinase K (Eurobio, France) for 8 min, washed with PBS, treated with 25 µl of *In Situ* Cell Death Detection mix (Roche, France) for three hours, and then washed with PBS and mounted in 25 µl of Vectashield Hardset containing 1.5 µg/ml DAPI (4′-6-Diamidino-2-phenylindole, Vector Laboratories, CA, USA). Positive and negative controls were treated by 50 U/µl DNAse (Fermentas, France) for 5 min before TUNEL staining, with or without TUNEL enzyme for the positive or negative controls, respectively. Preparations were observed using a fluorescence microscope (AxioCam Imager Z.1, Zeiss, Germany). A mean TUNEL index was built taking into account the location of the staining [germarium (G), close to germarium (CG), close to dumping (CD)] and its intensity [erratic points (EP×0.5), whole egg chamber (EC × n_EC_)], *i.e.* Tunel Index = 1_G_+n_EC/CG_+0.5_EP/CG_+n_EC/CD_+0.5_EP/CD_.

### Statistical analyses

When possible, parametric tests were used directly or after transformation of data. Expression data and iron absorption measurements were log-transformed and 1/x-transformed, respectively, before analysis. ANOVA residuals were checked for normality by Shapiro's test, and for homoscedasticity by Levene's test. Pairwise comparisons were then performed using Tukey's HSD test. To take into account all the experiments that were realized (2 blocks), number of wasps emerged and percentage of successful development of *Drosophila* were analysed using mixed Linear Model (LM) and mixed General Linear Model (GLM) with binomial error, respectively. In both cases, blocks were treated as random factor, whereas iron treatment and infection status were treated as fixed factors. All statistical analyses were performed using R 2.8.0 software.

## Supporting Information

Figure S1 Influence of *Wolbachia* infection and iron treatment on *D. simulans* survival. Survival curves of symbiotic and aposymbiotic females reared on standard diet (control) or iron-supplemented diet (n = 15 per treatment). Diet was renewed every 2 days and females were allowed to lay eggs.
(0.01 MB PDF)
