# Supplementary figures and images for: Wolbachia Interferes with Ferritin Expression and Iron Metabolism in Insects
Source: PLoS Pathog. 2009 Oct 23;5(10):e1000630. doi: 10.1371/journal.ppat.1000630 (PMC2759286; doi:10.1371/journal.ppat.1000630)

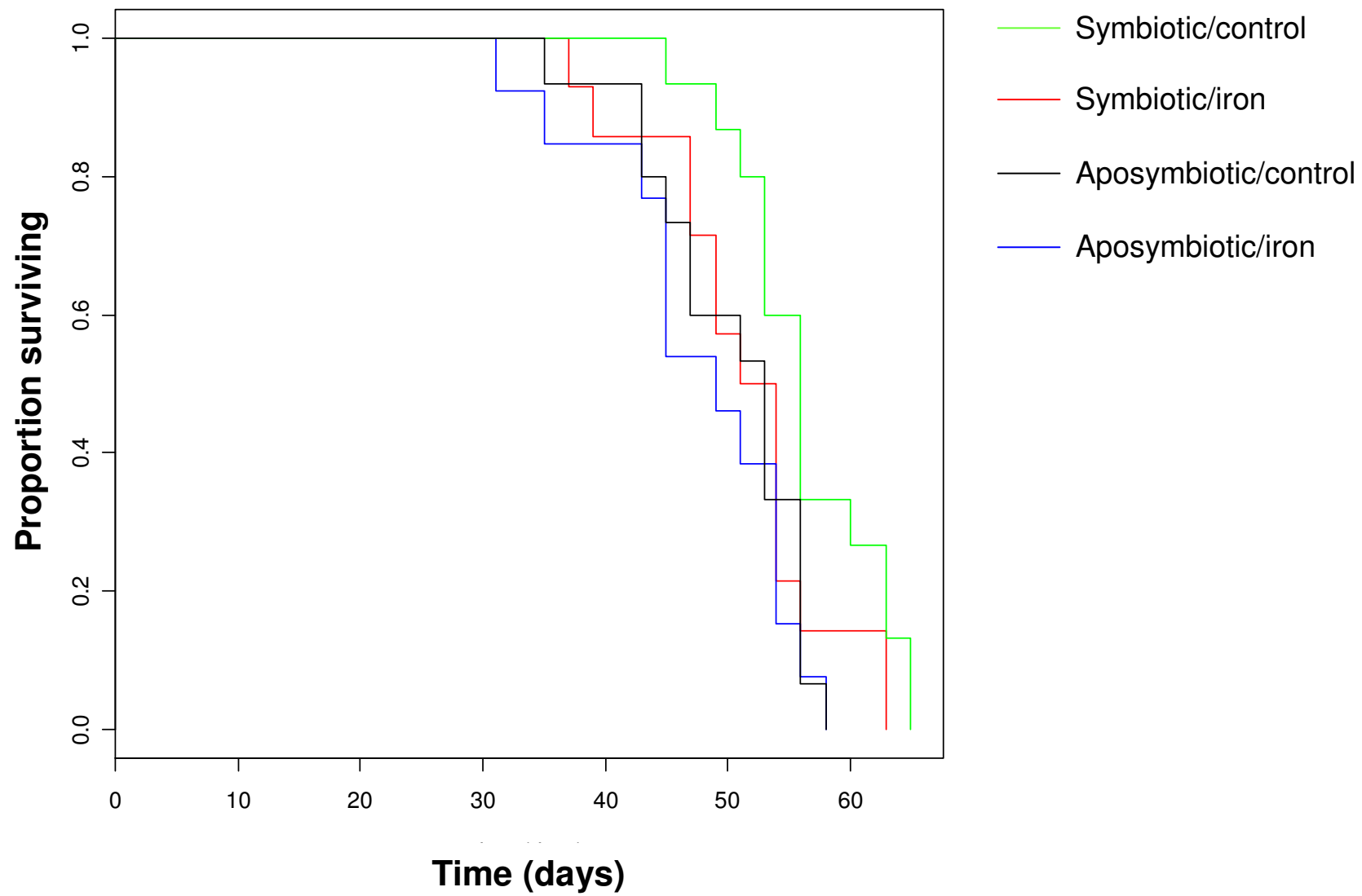

Supplement: Figure S1 — Influence of Wolbachia infection and iron treatment on D. simulans survival. Survival curves of symbiotic and aposymbiotic females reared on standard diet (control) or iron-supplemented diet (n = 15 per treatment). Diet was renewed every 2 days and females were allowed to lay eggs. (0.01 MB PDF) [file ppat.1000630.s001.pdf]
